# Effects of the Chinese Heart-Healthy Diet (Sichuan Cuisine Version) on the 10-year CVD risk and vascular age: a randomised controlled feeding trial

**DOI:** 10.1017/S0007114523002416

**Published:** 2024-03-28

**Authors:** Danping Su, Hong Chen, Yishan Guo, Qiuyu Feng, Mengtong Yang, Congjie Cai, Yiqi Zhang, Yangfeng Wu, Yanfang Wang, Guo Zeng

**Affiliations:** 1 Department of Nutrition and Food Safety, West China School of Public Health and West China Fourth Hospital, Sichuan University, Chengdu 610041, People’s Republic of China; 2 Peking University Clinical Research Institute, Peking University First Hospital, Beijing 100032, People’s Republic of China

**Keywords:** CVD, Cuisine culture, Healthy diet, Sichuan cuisine, Vascular ageing

## Abstract

Sichuan cuisine was previously fitted into the Chinese Heart-Healthy Diet (CHH) trial to verify the antihypertensive effect. Whether the modified Sichuan diet lessens cardiovascular disease (CVD) is not fully explored. We aimed to estimate the effects of the Sichuan version of CHH diet (CHH diet-SC) on the 10-year risk of CVD and vascular age. A single-blinded randomised controlled feeding trial was conducted. General CVD prediction model was used in manners of intention-to-treat and per-protocol set. After a 7-d run-in period, fifty-three participants with pre- and grade I hypertension from local communities were randomised and provided with either CHH diet-SC (*n* 27) or a control diet (*n* 26) for 4 weeks. Mean absolute and relative estimated CVD risks were reduced by 4·5 % and 27·9 % in the CHH diet-SC group, and the between-group relative risk reduction was 19·5 % (*P* < 0·001) using linear mixed-effects models. The sensitivity analysis with datasets and models showed consistent results, and pre-specified factors were not associated with the intervention effects. The vascular age of CHH-SC group was theoretically 4·4 years younger than that of the control group after intervention. Compared with a typical diet, adopting the CHH diet-SC over 1 month significantly reduced 10-year CVD risks and vascular ages among local adults with mild hypertension.

CVD is the leading cause of global mortality and is the foremost contributor to illness and death in China^(1)^. A suboptimal diet is one of the main reasons for the rapidly rising burden of CVD. China, one of the world’s twenty most populous countries, has the highest rate of diet-related CVD deaths^(2)^. A high-quality healthy diet has been viewed as both a modifiable risk factor and a vital lifestyle intervention strategy for CVD prevention^(3)^. Regularly monitoring CVD risk factors and assessing the general risk of CVD in a specific period of time (5 years, 10 years or lifetime) are jointly recommended by various countries for adults who are prone to CVD^(4–6)^. Risk estimation, as a method of primary care practice, can reflect the aggregation and progress of risk factors comprehensively.

A healthy dietary pattern for lowering CVD risk is encouraged in groups with or without CVD^(7)^. Dietary Approaches to Stop Hypertension (DASH)^(8)^, first proposed by the National Heart, Lung, and Blood Institute in 1997, has attracted much attention due to its initially distinct antihypertensive effect. Following such a diet has also been found beneficial for other CVD risk factors and metabolic-related diseases^(9–16)^. Specifically, previous studies^(17–19)^ have shown that high adherence to the DASH-style dietary pattern was associated with a reduced risk of specific CVD events. However, unifying a healthy DASH-style diet in massive regions of China can be challenging in consideration of the external difference of China – the West and the internal complexity of cuisine cultures. The Chinese Heart-Healthy Diet (CHH) trial^(20)^ has made the first attempt in reducing blood pressure across major cuisine cultures. Among them, Sichuan cuisine, originating from southwestern China, is the most distinctive and biggest cuisine with an international reputation for the multi-layered flavour and numb-spicy tastes, while its features of greasy dishes and salty condiments can be a potential barrier of health promotion and CVD prevention^(21)^.

The potential impact of a modified diet pattern based on Sichuan cuisine culture on overall CVD risks is unknown. Moreover, the additional benefits of such an intervention on abstract and visualised risks of CVD compared with a traditional diet have not been investigated. The objective of this study was to examine the impacts of short-term consumption of a CHH dietary pattern (Sichuan cuisine) for 4 weeks on the estimated 10-year general CVD risks. The laboratory version of the general CVD prediction model (GCVD) was used to estimate the 10-year CVD risk and to further calculate the vascular age as a risk-based approach for patients to better understand the abstract risk. We hypothesised that the CHH diet (Sichuan cuisine) would lower the absolute and relative 10-year CVD risk and would have an additional benefit in preventing CVD compared with a regular diet.

## Methods

### Study design and participant eligibility

**Fig. 1.**
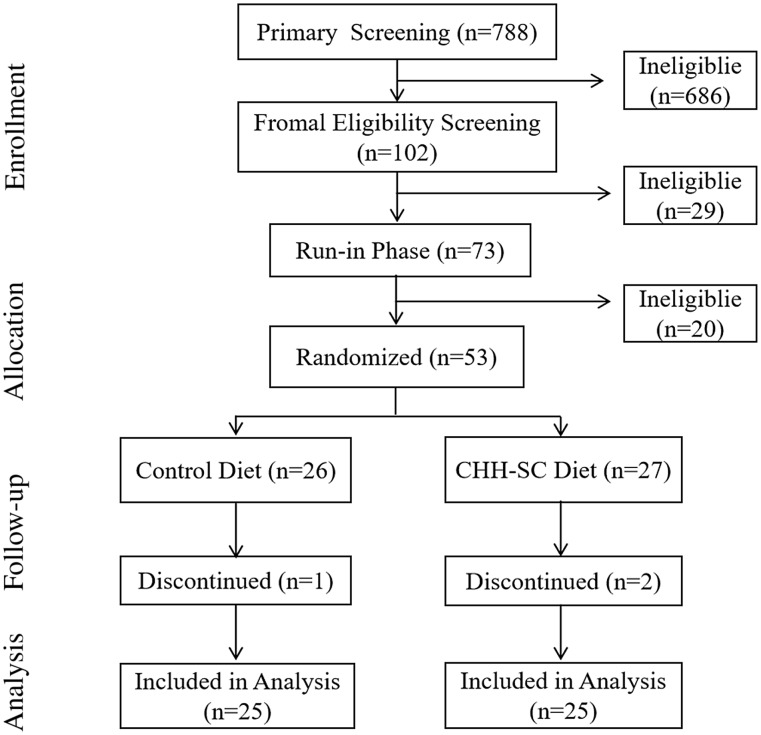
Flow diagram of the trial.

This secondary analysis of the CHH study used data in Chengdu, which is under the far-reaching influence of the Sichuan cuisine culture. The flow diagram of the Chengdu centre is shown in Fig. 1.

The details of the design and main results in reducing the blood pressure of the CHH study have been published elsewhere^(20)^. Shortly, this previous study was a multicentre (Beijing, Shanghai, Guangzhou and Chengdu), randomised controlled feeding trial. The main aim was to access the efficacy, preference and cost-effectiveness of the CHH diet. People aged 25–75 years old who were living in the local communities for at least 6 months before entering the study and had a systolic blood pressure (SBP) between 130 and 159 mmHg were eligible for this trial. More detailed inclusion and exclusion criteria were presented in the study design proposal^(22)^. The study protocol was first registered on the ClinicalTrials.gov (Unique identifier: NCT03882645) on 18 March 2019.

### Sample size

According to the findings from systematic reviews, the DASH diet can result in the reduction of the 10-year Framingham risk score for approximately 13 %. We conservatively assumed that the intervention diet will reduce relative risk by 7·0 % in comparison with the control diet in 4 weeks, with the standard deviation of risk score being 12·0 in the control group according to previous studies. To have 90 % power with a type I error rate of 5 % to detect the assumed effect size, we would need a total of sixty-two participants (thirty-one equally from each arm). Because of the COVID-19 epidemic and the quarantine policy, the study had to be terminated early, not achieving the planned sample size. Ultimately, we recruited a sum of fifty-three participants and reached 85·5 % of the goal setting.

### Run-in phase (1 week) and baseline assessment

Formally screened subjects received a 7-d run-in phase during which they were provided three meals per day for a total of twenty-one regular meals. Essentially, the run-in diet contained the feature of the common dietary pattern consumed by the local people following the previous food frequency survey and nutrition surveillance in Sichuan. Participants were asked of food preference and adjustments were made to best fit their preferred taste. A quantitative sample menu in the run-in period was attached to the supplementary files (online Supplementary Table 1). Subjects who failed to complete the run-in phase as required or ate less than 80 % of the study meals (at least eighteen meals) were excluded from the following randomisation. After completing the run-in phase, a self-designed questionnaire was administered in face-to-face interviews to collect baseline information about general demographic data, lifestyle, dietary habits, history of illness and medication information.

### Random assignment and blinding

Participants who passed the run-in phase were randomly assigned to the CHH-SC group and the control group by a computer at a ratio of 1:1. Due to the participant-blinded design, the intervention and control groups ate at separate close-door rooms without being informed about their group assignment. Similar menus and interchangeable ingredients were provided in the same meal. Trained staff conducted blood pressure measurements or collecting the laboratory measurements and cooks were also masked in the assignment.

### Interventions (4 weeks)

All participants were requested to consume the corresponding three meals (breakfast, lunch and dinner) per day for the following 4 weeks. No other interventions were employed in either group. All study meals were prepared in standardised research kitchens and consisted of available and interchangeable foods on the premise of meeting the daily nutrient and energy composition targets. It encompassed non-repetitive menus within a cycle of 2 weeks, designed jointly by the study nutritionists, dietitians and chefs. Quantitative sample dishes in both groups were attached to the supplementary files (online Supplementary Tables 2 and 3).

Participants assigned to the control group continued with the regular diet as in the run-in phase. The control diet-SC was a typical diet of Sichuan residents, based on previous surveys of usual local foods. It was adjusted to the common flavour and taste during the run-in period for higher adherence.

The CHH diet-SC was a healthy dietary pattern based on Sichuan cuisine culture (e.g. the featured aromas of mixed seasonings). The nutrients targets and primary components for dietary classes were developed by integrating information from the Chinese Dietary Guidelines, previous healthy dietary patterns, Dietary Consumption Survey of Sichuan area and the most common Sichuan cuisine dishes. Shortly, it modified some key dietary factors (i.e. whole grains, fresh fruits and vegetables, processed meat, low-fat dairy products, cooking oil and salt) as regards healthy eating patterns, among which, researches indicate that the DASH diet exhibits a substantial capacity in lowering blood pressure and providing safeguarding benefits on other CVD risk factors. The main cooking method consisted of reducing cooking oil and salt by using natural flavours (such as coriander and star anise) and using low-sodium salt.

### Dietary assessments

During the whole intervention, we requested participants to consume the dishes we provided at lunch and dinner on site while the breakfast was distributed in advance at dinner the day before. The leftovers and supplements of each meal and all amounts of snacks outside the design were precisely assessed by the trained staff through weighing or validated visual estimation by the help of illustrative food pictures albums.

Daily food intake and nutrients were appraised using the nutrition calculator with the built-in China Food Composition Table (2018) and Dietary Reference Intakes for Chinese (2013). According to the Food Guide Pagoda of Chinese Dietary Guidelines (2022), items of food groups and corresponding food subgroups were classified to assess the food intake of the CHH-SC dietary pattern. Food variety was calculated by summing the number of unique food items with a minimum intake of 10 g without cooking oils and condiments. Estimations of fatty acids and vitamin B_12_ were further supplemented with the United States Department of Agriculture Nutrient database.

### Measurements of dietary adherence of participants and CVD risk factors

All measurements conducted at baseline were repeated at the end of the trial. In addition, blood pressure, body weight and food preference score were measured weekly to track the trial implementation.

For assessment of food preference, participants were asked at the end of each week to rate their preference of the previous week’s meals on a visual analogue scale ranging from 0 to 10 (10 represents most preferred).

Height and body weight were measured using a standard protocol and used to calculate BMI. Morning blood pressure was measured three times within a day (08.00–10.00, 14.00–16.00 and 06.00–20.00 hours), each time for three readings, using an Omron HEM-7136 blood pressure monitor (Omron Health Care Co, Ltd). A total of nine readings at baseline and at the end of study were averaged and used in the analysis.

Morning fasting blood samples and urine samples were collected and sent for analysis to a central laboratory. Total cholesterol (TC), LDL-cholesterol, HDL-cholesterol, triglyceride (TG) and urinary electrolytes were quantified from samples. The definition of dyslipidaemia at baseline was according to the 2016 Chinese guidelines for the management of dyslipidaemia in adults^(23)^.

Considering 24-h urine sodium as the gold standard for assessing salt intake, we transferred single-point urinary output into 24-h urinary output using the method developed in the China Salt Substitute and Stroke Study^(24)^, uncorrected for urine creatinine. The estimated 24-h urine sodium equals (individual volume spot sodium concentration in individual spot urine sample) × (volume of an individual 24-h urine sample), where the mean sample volume was 2·5 l from the first batch of urine samples.

### CVD risk identification and vascular age

The general CVD prediction model (laboratory version)^(25)^, developed from the Framingham Heart Study, was generated using a sex-stratified Cox proportional hazards regression to predict individual overall CVD events (mainly CHD, stroke, peripheral artery disease or heart failure). Sex, age, diabetes, smoking status, treatment for hypertension, SBP, TC and HDL were as risk factors involved in the laboratory-based version. Smoking status, diabetes and sex were entered as binary variables, while age, TC, HDL and non-treated/treated SBP were entered as sequential components. To quantify the CVD risk, we first established the major CVD risk factors in our study. TC, HDL and SBP measured at baseline and after a 4-week intervention were mainly used while other components of the model were collected at baseline. Then, we used the general formula calculation and the score sheet to assess an individual’s 10-year total CVD risk score according to the layered sex.

Another sex-specific multivariable risk algorithm, the Globorisk model (laboratory version)^(26)^, developed by Harvard researchers and recalibrated by Chinese cohorts population, was used in our sensitivity analyses. In this model, fatal and non-fatal CVD rates were calculated as fatal ischaemic heart disease + fatal stroke + (1 - (1 – non-fatal ischaemic heart disease) × (1 - non-fatal stroke)), allowing for the intending overlap between non-fatal ischaemic heart disease and stroke. All calculation formula above was described in the corresponding references.

The absolute CVD risk of an individual was further transformed into the vascular age and lost age in the same GCVD prediction model. Vascular age is a risk-based term of the individual’s age with the same risk score but all risk factors at the normal level. And lost age^(27)^ was the loss of vascular age adjusted for chronological age, implying the ongoing severity of vascular ageing. For example, a 50-year-old man with a 65-year-old vascular age was predicted to have lost years of 15.

### Statistical analyses

We analysed our data using IBM SPSS Statistics for Windows Version 27.0 (IBM Corp.). Means, sd or 95 % CI are presented for continuous variables, and proportion and frequency are used to describe qualitative data. We conducted a comparison of the demographic characteristics of participants using the *χ* ^2^ test or independent *t* test in accordance with categorical and continuous variables. For quantitative normally and abnormally distributed data, a paired *t* test or a paired Wilcoxon test was used for within-group comparisons. To estimate the intervention effects, linear mixed-effects models with the restricted maximum likelihood method were used to examine differences in pre- and post-intervention changes in the outcomes of two groups. In the linear mixed-effects models, risk values were entered as dependent variable, and the fixed portion of the models included diet to get the between-participant intervention effect and time-point to get the within-participant effect. A diet-by-visit interaction term was evaluated to assess the extent of differences between groups over time. The random-effects portion of the model included participant identification with a random intercept. The models were also adjusted for the weight and the baseline risk score to correct the weight changes and differences in baseline value.

Primary analyses of CVD risk estimation were restricted to the per-protocol set data with complete blood pressure and blood lipid data. Mean CVD risk scores at baseline and the 4-week visit were calculated for each group. The primary outcomes were the absolute and relative difference in the estimated 10-year CVD risk score based on SBP, TC and HDL from baseline to the end of the trial. For ease of interpretation, we used the absolute risk reduction to compare the individual’s net change of difference and the number needed to treat (NNT), the inverse of absolute risk reduction, to assess the value of promoting such a diet to prevent one CVD event. The relative risk reduction (RRR) was then used to explore relative changes from baseline to the end of the trial relative to the baseline level. RRR equals the baseline risk score minus the final risk score divided by the baseline risk score.

In sensitivity analysis, the estimated 10-year CVD risks were recalculated separately for the full analysis set and the Globorisk model. The multiple imputation for continuous variables by chained equations was used to replace the missing values in the full analysis set. Agreement between the two methods was assessed using the intra-class correlation coefficient to confirm the robustness of the results among GCVD and Globorisk.

In the exploratory analysis, we evaluated interactions between the interventions and subgroups categorised by baseline factors (sex, age (< 65 years or ≥ 65 years), BMI (< 24·0 or ≥ 24·0 kg/m^2^), history of hypertension (yes or no), treatment for hypertension (yes or no), dyslipidaemia status (yes or no), baseline SBP (< 140 mmHg or ≥ 140 mmHg) and baseline CVD risk (< 20 % or ≥ 20 %)). We included a main effect for the interventions and interactions between the subgroup and the interventions. In addition, based on the risk estimation, the between-group differences in changes in vascular ages and lost ages were further examined.

## Results

### Baseline demographic characteristics

A total of fifty-three subjects were randomised into either the control group (*n* 26) or the CHH-SC group (*n* 27). The baseline characteristics of two groups are shown in Table 1, with baseline comparability.

**Table 1.** Baseline characteristics of participants (Numbers and percentages; mean values and standard deviations)

	Control-SC (*n* 26)		CHH-SC (*n* 27)			Total (*n* 53)	
Characteristic	*n*	%	*n*	%	*P* *	*n*	%
Age, years							
Mean	60·7		59·8		0·712	60·2	
sd	9·3		9·5			9·3	
Male	17	65·4	11	40·7	0·072	28	52·8
Married	21	80·8	23	85·2	0·728	44	83·0
Household income (USD/month)†					0·64		
$ 2900–7250	2	7·7	1	3·7		3	5·7
$1450–2900	5	19·2	8	29·6		13	24·5
< $ 1450	19	73·1	18	66·7		37	69·8
Occupation					0·757		
Manual labourers	15	57·7	15	55·6		30	56·6
Housekeeping	9	34·6	11	40·7		20	37·7
Others	2	7·7	1	3·7		3	5·7
Education					0·268		
Bachelor’s degree or college and above	5	19·2	2	7·4		7	13·2
High school or technical secondary school	4	15·4	7	25·9		11	20·8
Junior high school or less	17	65·4	18	66·7		35	66·0
Medical insurance					0·139		
Employee or resident medical insurance	21	80·8	18	66·7		39	73·6
New rural cooperative medical insurance‡	3	11·5	8	29·6		11	20·8
Others	2	7·7	1	3·7		3	5·7
Current smoking	6	23·1	4	14·8	0·501	10	18·9
Alcohol	5	19·2	2	7·4	0·25	7	13·2
Physical activity level§					0·491		
Inadequate	7	25·9	9	34·6		16	30·2
Adequate	20	74·1	17	65·4		37	69·8
Hypertension	17	65·4	19	70·4	0·697	36	67·9
Treatment for hypertension	12	46·2	15	55·6	0·494	27	50·9
Overweight and obese||	21	80·8	23	85·2	0·728	44	83·0
	Mean	sd	Mean	sd		Mean	sd
Baseline BMI, kg/m^2^	25·8	2·2	25·9	2·2	0·928	25·9	2·2
Baseline SBP, mmHg	140·2	6·7	140·4	8·3	0·912	140·3	7·5
Baseline DBP, mmHg	88·3	7·7	87·8	8·6	0·835	88·0	8·1

CHH-SC, Sichuan version of Chinese Heart-Healthy Diet; SBP, systolic blood pressure; DBP, diastolic blood pressure.

* Independent *t* test and *χ* ^2^ test for continuous and categorical variables, respectively.

† 1 CNY = 0·1450 USD.

‡ New rural cooperative medical insurance is a type of rural cooperative medical insurance based on overall planning for serious diseases.

§ According to the Chinese Dietary Guidelines (2022) recommendations on physical activity, a total of 150 min or more of moderate-intensity physical activity per week is classified as ‘adequate’.

|| Overweight: 24·0 ≤ BMI < 28·0; obese: BMI ≥ 28·0.

### Dietary characteristics

Table 2 displays the dietary analysis of food groups and subgroups for both groups. After 4 weeks of intervention, we found a higher increase (*P* < 0·001) in dietary intakes of total fruits, dairy products, soyabeans and nuts with the CHH-SC group compared with the control group. Conversely, the daily intakes of cooking oil (26·1 g/d *v*. 42·2 g/d) and salt (4·0 g/d *v*. 7·1 g/d) in the CHH-SC group were approximately half of those in the control group.

**Table 2.** Daily average intake of food groups and diversity in 4 weeks, by CHH diet-SC and control diet-SC

Variables	CHH-SC, mean	Control-SC, mean	Mean difference	95 % CI	*P* *
Daily food (g)					
Cereals	381·2	369·2	12·0	–8·5, 32·5	**0·250**
Whole cereals	142·8	38·7	104·2	86·7, 121·6	**< 0·001**
Tubers	17·6	46·2	–28·6	–45·3, −11·9	**0·001**
Vegetables	434·4	452·1	–17·7	–45·2, 9·8	0·205
Stem, leafy and flowering	289·6	211·3	78·2	47·7, 108·8	**< 0·001**
Fruits	251·6	82·1	169·5	141·3, 197·7	**< 0·001**
Kernel fruits	170·1	49·8	120·3	95·2, 145·4	**< 0·001**
Animal food	205·3	203·0	2·3	–8·3, 12·9	0·669
Red meat	81·4	130·8	–49·5	–58·2, −40·7	**< 0·001**
Seafood	28·9	9·4	19·5	9·8, 29·2	**< 0·001**
Eggs	42·7	29·1	13·6	4·0, 23·3	**0·006**
Dairy products†	269·5	75·6	193·9	166·2, 221·6	**< 0·001**
Soyabeans and nuts	43·6	19·9	23·7	17·7, 29·8	**< 0·001**
Cooking oil	26·1	42·2	–16·1	–17·9, −14·4	**< 0·001**
Salt‡	4·0	7·1	–3·1	–3·5, −2·7	**< 0·001**
Food variety§	18·3	13·5	4·8	4·2, 5·4	**< 0·001**

CHH diet-SC, Sichuan version of Chinese Heart-Healthy Diet.

* Bold values were statistically significant.

† Low fat (2 %) in the CHH-SC diet.

‡ Low sodium (76 %) in the CHH-SC diet; normal sodium (> 99·9 %) in the control diet.

§ Number of different food items consumed by participants. Condiments and cooking oil were excluded. Accounting for food items with > 10 g.

Regarding food subgroups, whole cereals, stem, leafy and flowering vegetables, kernel fruits and seafood were consumed more, while red meat and tubers were restricted in the CHH-SC group. For both groups, the daily intakes of important nutrient components in a healthy dietary pattern are presented in Table 3.

**Table 3.** Daily average intake of nutrients in 4 weeks, by CHH diet-SC and control diet-SC (95 % confidence intervals)

Nutrients	CHH-SC, mean	Control-SC, mean	Mean difference	95 % CI	*P* *
Energy (kcal/d)	2253·0	2400·2	–147·3	–195·6, −98·9	**< 0·001**
Carbohydrate (% E)	57·3	52·8	4·6	3·5, 5·6	**< 0·001**
Protein (% E)	17·9	12·9	5·0	4·5, 5·5	**< 0·001**
Total fat (% E)	24·8	34·4	–9·6	–10·7, −8·6	**< 0·001**
SFA	6·2	9·6	–3·4	–3·8, −3·0	**< 0·001**
MUFA	7·5	12·9	–5·4	–6·0, −4·8	**< 0·001**
PUFA	8·9	9·1	–0·2	–0·6, 0·2	0·329
Fibre (g)	29·5	13·8	15·7	14·2, 17·2	**< 0·001**
Cholesterol (mg)	412·2	340·2	72·0	22·4, 121·6	**0·005**
Na (mg)	2492·5	5898·5	–3405·9	–4177·3, −2634·6	**< 0·001**
K (mg)	4108·7	2357·3	1751·4	1530·9, 1972·0	**< 0·001**
Ca (mg)	980·3	420·9	559·5	520·6, 598·3	**< 0·001**
Mg (mg)	550·4	316·2	234·2	210·9, 257·5	**< 0·001**
Se (mg)	47·6	36·4	11·2	7·8, 14·7	**< 0·001**
Vitamin A (μgRAE)	1047·8	668·1	379·7	60·6, 698·8	**0·020**
Vitamin E (mg)	46·9	40·7	6·3	3·6, 9·0	**< 0·001**
Vitamin C (mg)	152·2	120·7	31·5	15·5, 47·4	**< 0·001**
Folic acid (μg)	266·3	184·1	82·1	47·0, 117·2	**< 0·001**
Vitamin B_12_ (μg)	6·5	3·8	2·6	1·4, 3·9	**< 0·001**

CHH diet-SC, Sichuan version of Chinese Heart-Healthy Diet; SFA, saturated fatty acids; MUFA, monounsaturated fatty acids; PUFA, polyunsaturated fatty acids.

* Bold values were statistically significant.

### Dietary adherence of participants and changes in CVD risk factors

Of the randomised participants, 94·3 % completed the trial (50/53). All three participants who discontinued our trial chose to terminate the trial for personal reasons. The dietary adherence of participants was assessed using both subjective and objective measures (Table 4). Food preference scores were close to 10 points and were similar in the two groups (*P* = 0·841). Weight loss was controlled within 2 kg, consistent with the study protocol. The trends of 24-h urinary sodium excretion were in line with changes in dietary sodium intakes.

**Table 4.** Dietary adherence and CVD risk factors, by CHH diet-SC and control diet-SC (Mean values and standard deviations; 95 % confidence intervals)

Variables		CHH-SC (*n* 25)		Control-SC (*n* 25)		Difference in difference	95 % CI	*P* ‡
		Mean	sd	Mean	sd			
Food preference	Baseline	9·7	0·5	9·6	0·5			
	4 weeks	10·0	0·0	9·9	0·2			
	Difference	0·3	0·5	0·3	0·5	0·0	–0·3, 0·3	0·841
Weight (kg)	Baseline	64·2	8·5	64·0	7·8			
	4 weeks	63·1	8·6	63·6	7·6			
	Difference	–1·1	1·0	–0·5	1·0	–0·7	–1·2, −0·1	**0·022**
UNa (mg/24 h)	Baseline	6741·3	2911·8	7070·2	2594·4			
	4 weeks	4393·0	2214·2	7337·0	2681·8			
	Difference	–2348·3	2397·2	266·8	3042·8	–2615·1	–4172·8, −1057·4	**0·001**
SBP (mmHg)	Baseline	141·0	8·3	140·2	6·8			
	4 weeks	125·8	9·6	135·2	11·4			
	Difference	–18·2	9·0	–9·3	12·1	–8·8	–14·9, −2·7	**0·005**
DBP (mmHg)	Baseline	88·1	8·7	88·1	7·8			
	4 weeks	82·5	8·5	85·8	8·8			
	Difference	–8·6	6·4	–4·2	6·2	–4·5	–8·1, −0·9	**0·015**
TC (mg/dl)*	Baseline	175·7	33·7	180·5	33·8			
	4 weeks	159·4	27·5	168·5	35·0			
	Difference	–16·3	17·3	–12·0	22·0	–4·3	–15·6, 6·9	0·440
LDL (mg/dl)*	Baseline	100·4	29·5	98·9	32·0			
	4 weeks	90·7	23·3	89·8	24·0			
	Difference	–9·8	15·8	–9·1	20·1	–0·7	–11·0, 9·6	0·895
TG (mg/dl)†	Baseline	144·2	63·0	148·4	102·1			
	4 weeks	125·9	44·5	143·7	87·5			
	Difference	–18·3	39·7	–4·6	34·5	–13·7	–34·8, 7·5	0·200
HDL (mg/dl)*	Baseline	44·1	7·5	48·6	12·1			
	4 weeks	43·1	7·6	46·3	11·2			
	Difference	–2·0	3·7	–2·3	4·3	0·3	–2·0, 2·6	0·808

CHH diet-SC, Sichuan version of Chinese Heart-Healthy Diet; SBP, systolic blood pressure; DBP, diastolic blood pressure; UNa, urinary sodium excretion; TC, total cholesterol; TG, triglyceride.

* 1 mmol/l = 38·7 mg/dl.

† 1 mmol/l = 88·6 mg/dl.

‡ Bold values were statistically significant.

Table 4 also displays the changes in CVD modifiable risk factors (blood pressure and blood lipids) in this trial. Significant intervention effects were observed for blood pressure. The CHH diet-SC also resulted in slight but insignificant improvements in the mean serum concentrations of blood lipids compared with the control diet.

### Estimated 10-year CVD risks and vascular ages

Table 5 shows that with comparable baseline risk, the within-group risk of CVD was absolutely/relatively reduced by 1·6 %/8·4 % and 4·5 %/27·9 % in the control and intervention groups, respectively, at 4 weeks. The net difference in absolute risk reduction between the CHH diet-SC and the control diet-SC was 2·9 % (95 % CI 1·1, 4·7; *P* = 0·002). The number of NNT was 22 in the intervention group, while it almost tripled (NNT = 63) in the control group. The between-group changes in RRR were 19·5 % (95 % CI 9·6, 29·4; *P* < 0·001), representing a significant reduction in the relative risk of CVD in the CHH-SC group compared with the control group over 4 weeks. Table 5 also shows that the results from the analysis of the full analysis set dataset were virtually identical.

**Table 5.** Estimated 10-year CVD risk (%), by CHH diet-SC and control diet-SC (Mean values and standard deviations; 95 % confidence intervals)

Variables	CHH-SC		Control-SC		Adjusted Mean Difference*	95 % CI	*P* †
	Mean	sd	Mean	sd			
Using general CVD profile – PPS							
Baseline	16·6	11·2	21·7	11·3			0·118
4 weeks	12·1	9·0	20·1	11·7			
ARR	4·5	3·1	1·6	3·4	2·9	1·1, 4·7	**0·002**
RRR	27·9	12·3	8·4	21·3	19·5	9·6, 29·4	**< 0·001**
NNT, *n*	22		63				
Using general CVD profile – FAS							
Baseline	16·8	11·5	21·1	11·4			0·173
4 weeks	12·6	9·5	19·7	11·6			
ARR	4·2	3·2	1·4	3·5	2·6	1·1, 4·0	**0·001**
RRR	25·7	14·5	6·0	24·2	19·7	8·7, 30·6	**0·001**

CHH diet-SC, Sichuan version of Chinese Heart-Healthy Diet; PPS, per-protocol set; FAS, full analysis set; ARR, absolute risk reduction; RRR, relative risk reduction; NNT, number needed to treat.

* All models adjusted for weight change and baseline risk score.

† Bold values were statistically significant.

Moreover, the models showed relatively good agreement between the risk scores, and the intra-class correlation coefficient was estimated to be 0·860 (95 % CI 0·770, 0·916) in men and 0·637 (95 % CI 0·428, 0·782) in women (not shown in tables). No interaction effect was observed across subgroups for CHH-SC *v* the control group (*P* all > 0·05) (Table 6).

**Table 6.** Relative reduction (mean, 95 % CI) in 10-year CVD risk score in strata of baseline information (Numbers and 95 % confidence intervals)

Characteristic	*n*	Mean difference	95 % CI	*P*_interaction_
Sex				0·387
Male	27	13·2	6·5, 19·9	
Female	23	24·0	14·6, 33·4	
Age (years)				0·936
< 65	29	17·1	8·5, 25·8	
≥ 65	21	19·6	12·7, 26·6	
Baseline BMI (kg/m^2^)				0·489
< 24·0	10	13·6	2·7, 24·4	
≥ 24·0	40	19·3	12·7, 26·0	
Hypertension				0·902
No	15	18·2	8·2, 28·2	
Yes	35	18·2	11·0, 25·3	
Treatment for hypertension				0·372
No	23	18·9	11·6, 26·2	
Yes	27	17·5	8·7, 26·4	
Dyslipidaemia				0·958
No	32	14·4	6·9, 21·8	
Yes	18	25·0	16·8, 33·3	
Baseline SBP (mmHg)				0·224
< 140	25	16·1	9·1, 23·1	
≥ 140	25	20·3	11·0, 30·0	
Baseline CVD risk (%)				0·78
< 20	31	20·2	12·3, 28·1	
≥ 20	19	14·8	6·8, 22·9	
Overall	50	18·2	12·5, 23·8	

SBP, systolic blood pressure.

Furthermore, the changes in vascular age were proven to be effective in the CHH-SC group relative to the control diet-SC (*P* = 0·004), with saving the lost age of 3·6 years (95 % CI −6·3, −0·9) (Table 7).

**Table 7.** Chronological/vascular age and lost age, by CHH diet-SC and control diet-SC (Mean values and standard deviations; 95 % confidence intervals)

Variables	CHH-SC (*n* 25)		Control-SC (*n* 25)		Difference in difference	95 % CI	*P* *
	Mean	sd	Mean	sd			
Chronological age (years)	60·3	9·5	61·1	9·4			0·766
Vascular age (years)							
Baseline	68·6	12·8	70·7	10·6			0·541
4 weeks	62·7	13·7	69·2	12·5			
Difference	–5·9	5·1	–1·5	5·4	–4·4	–7·4, −1·5	**0·004**
Lost age (years)							
Baseline	8·7	6·3	10·4	8·0			0·404
4 weeks	4·4	6·0	9·4	8·9			
Difference	–4·3	4·8	–0·7	4·8	–3·6	–6·3, −0·9	**0·010**

CHH diet-SC, Sichuan version of Chinese Heart-Healthy Diet.

* Bold values were statistically significant.

## Discussion

Overall, our study indicated the potential benefits of the Sichuan cuisine version of CHH diet in lowering the risk of 10-year general CVD events in a group of adults with pre- or grade I hypertension. The CHH diet-SC significantly reduced the estimated 10-year overall CVD risk by 4·5 % (RRR of 27·9 %), with a between-group difference in absolute risk reduction of 2·9 % (RRR: 19·5 %) over 4 weeks. The results were identical in sensitivity analyses using different datasets and prediction models. Among subgroups defined by baseline information, the results of estimated risks were similar. In addition, our study converted the abstract risks to vascular ages contributing to a better understanding of CVD risk from the patient side and still found nearly 4 years were regained after adjusting for chronological ages.

One explanation of the protective association between CHH diet-SC and CVD is that dietary adherence to DASH provided certain evidence of the potential benefits for cardiovascular health. Previous studies to investigate the effects of DASH on specific CVD events have commonly used various risk assessment models. Using the Framingham Stroke Risk Profile, Chen *et al*.^(28)^ documented that the original DASH diet reduced the estimated 10-year coronary heart disease (CHD) risk by 18 % compared with the control group. Framingham Stroke Risk Profile produced sex-specific prediction for risks of developing incident CHD in a white middle-class population^(29)^. However, for Asians, the original Framingham Stroke Risk Profile overestimated the risk of CHD and needed recalibration to improve the estimations^(30)^. The new research^(31)^ used the American College of Cardiology/American Heart Associated Pooled Cohort Equation, suggesting that compared with a typical American diet, the DASH diet reduced 10-year arteriosclerotic CVD risk scores by approximately 10 % in 8 weeks. The variation of effect estimations may be attributed to the model heterogeneity and the interdietary characteristics. In contrast, another DASH-related randomised controlled trial (RCT)^(32)^ adapted the Chinese recalibration of Framingham Stroke Risk Profile and found no significant between-group difference in the reduction in 10-year CHD risk at 6 and 12 months. This finding reflected the possible restriction of the dietary impact on lowering the CVD risk in a clinical setting. Longer follow-up visits are warranted in a real-world setting.

The sensitivity analyses of the consistency towards the results from GCVD and Globorisk models for predicting CVD risks further verified the moderate stability of the association between Sichuan-cuisine-based dietary intervention and lowering CVD risks. Globorisk has modified by the Chinese adult population where China Health and Nutrition Survey analysis files provided the prevalence estimates of total CVD risk.

The NNT in our study, as a quantitative parameter of the academic efficacy of the CHH-SC dietary intervention, allowed health practitioners to be aware of the concept of how much effort is needed to avoid a general CVD event. Our study also extended the literature in the fields of dietary intervention and nutritional counselling by investigating the effect of CHH diet-SC on vascular age. How to improve patients’ self-management awareness and treatment adherence is a plight for clinical practice when it comes to risk-based estimates. Comparing with informing patients about their risks, expressing as the vascular age is viewed as an ideal approach for achieving better communication and higher treatment adherence among patients who have a higher baseline risk of CVD but have not yet developed an actual CVD event^(27,33)^. The CHH diet-SC is valid as a changeable part of primary health care for large amount of population who get used to Sichuan cuisine widely. Unexpectedly, due to the study sample size, our findings about NNT and Vascular Age tool should be considered exploratory rather than confirmatory.

The effect of the CHH diet-SC might be attributed to the advantageous combination of modified food groups and nutrients. The CHH diet-SC is a food-diverse pattern, high in whole cereals, vegetables (in particular stem, leafy and flowering vegetables), fruits and low-fat dairy products, with restrictions on red meat, cooking oil and salt. Current studies have shown that whole grains can help prevent CHD, CVD and all-cause mortality. The relative risk per 90 g/d increase in whole grain intake was 0·78 (95 % CI 0·73, 0·85) for CVD^(34)^. Furthermore, diets rich in vegetables and fruits might prevent the morbidity and mortality of CVD according to several prospective cohort studies^(35)^. Among vegetable sources, green leafy vegetables provided greater benefits, whereas kernel fruits provided greater benefits among fruit sources. An inverse association between diet diversity and CVD incidence was also observed among non-Europeans consuming diverse food groups^(36)^. In addition, the consumption of low-fat dairy products, less red meat and less cooking oil in the CHH diet-SC limited the intake of SFA. Overt risk reduction by 17 % (relative risk: 0·83; 95 % CI 0·70, 0·98) in combined cardiovascular events potentially resulted from reducing SFA from systematic reviews^(37)^. Moreover, a significant linear relationship between dietary sodium intake and CVD risk was found in a dose-response meta-analysis^(38)^. The characteristic of high potassium/low sodium in the CHH diet-SC mechanically supported our results.

Considering the included CVD factors, except for the obvious antihypertensive effect, our study found slight but insignificant advantages of CHH-SC on the concentration of blood lipids. Another meta-analysis^(39)^ of twenty good-quality RCT showed a significant decrease caused by the DASH diet in several cardio-metabolic biomarkers, including SBP, diastolic blood pressure, TC and LDL. However, these interventions did not affect TG, HDL or glucose. In addition to the short-term intervention, the explanations at the nutrient level might include the macronutrient pattern. From a meta-analysis of 121 eligible trials^(40)^, neither DASH (moderate macronutrient pattern) nor low-fat (≤ 30 % fat) dietary patterns were superior to the usual diet in terms of blood lipoproteins.

A major strength of our study is the strong internal validity with the strict design of a controlled and randomised feeding trial, the standardised assessments and the high adherence of participants. The feeding design, as well as the quantitative leftovers and snacks, ensured that the assessment of dietary intake was as precise as possible. Considering both the burden of CVD and unbalanced features of the usual diet (e.g. high-fat and salty dishes), another strength is that our study made translation efforts to implement healthy dietary patterns and cater to domestic cuisine culture to prevent CVD. Moreover, few studies of dietary intervention have used general CVD event risk as an outcome variable. To date, we are sure that this is innovative to test the effects of a cuisine-culture-based healthy dietary pattern on predicting total CVD outcomes and further visualise the risk as vascular age using the GCVD prediction model.

Notably, our study was subject to several limitations. First, although common for a feeding trial, the sample size was not powered to analyse all subgroups. Second, the 4-week intervention was too short to observe adherence in the real world due to the cost restriction. Nevertheless, long-term randomised trials with true CVD endpoints were not feasible for most dietary factors. Third, our results of estimated 10-year general CVD risk were assessed by the prediction model and were not the actual CVD events. Thus, our conclusions should be extrapolated to long-term, real-world situations with caution. Fourth, our regional hub-data reflected the lack of representativeness of the population. Future research should focus on establishing the various components that are both most effective in reducing CVD risk and most representative of Sichuan dishes. The challenge of implementing the CHH diet-SC in a real-world setting cannot be underestimated. If possible, the actual CVD event incidence observed in follow-up visits should also be compared with the estimated CVD risk as a surrogate outcome.

In conclusion, the CHH diet-SC, tailored to local cuisine features, has been proven to significantly lower the absolute and relative estimated 10-year general CVD risk by 2·9 % and 19·5 % and has a decrement of around 4-year vascular age in 1 month, relative to a regular diet. Furthermore, our study provided evidence to encourage further investigation of healthy diet patterns to support groups exposed to multiple CVD risk factors.

## Supporting information

Su et al. supplementary material Su et al. supplementary material

## Supplementary material

For supplementary material referred to in this article, please visit https://doi.org/10.1017/S0007114523002416
