# Supplementary material for: Effects of the Chinese Heart-Healthy Diet (Sichuan Cuisine Version) on the 10-year CVD risk and vascular age: a randomised controlled feeding trial
Source: Br J Nutr. 2023 Nov 6;131(6):997–1006. doi: 10.1017/S0007114523002416 (PMC10876454; doi:10.1017/S0007114523002416)
Supplement: Su et al. supplementary material [file S0007114523002416sup001.docx]

**Table S1** A quantitative sample menu in 1-week run-in period (2150 kcal/d)

| Meals | Dishes | Ingredients | Weights (g) |
| --- | --- | --- | --- |
| **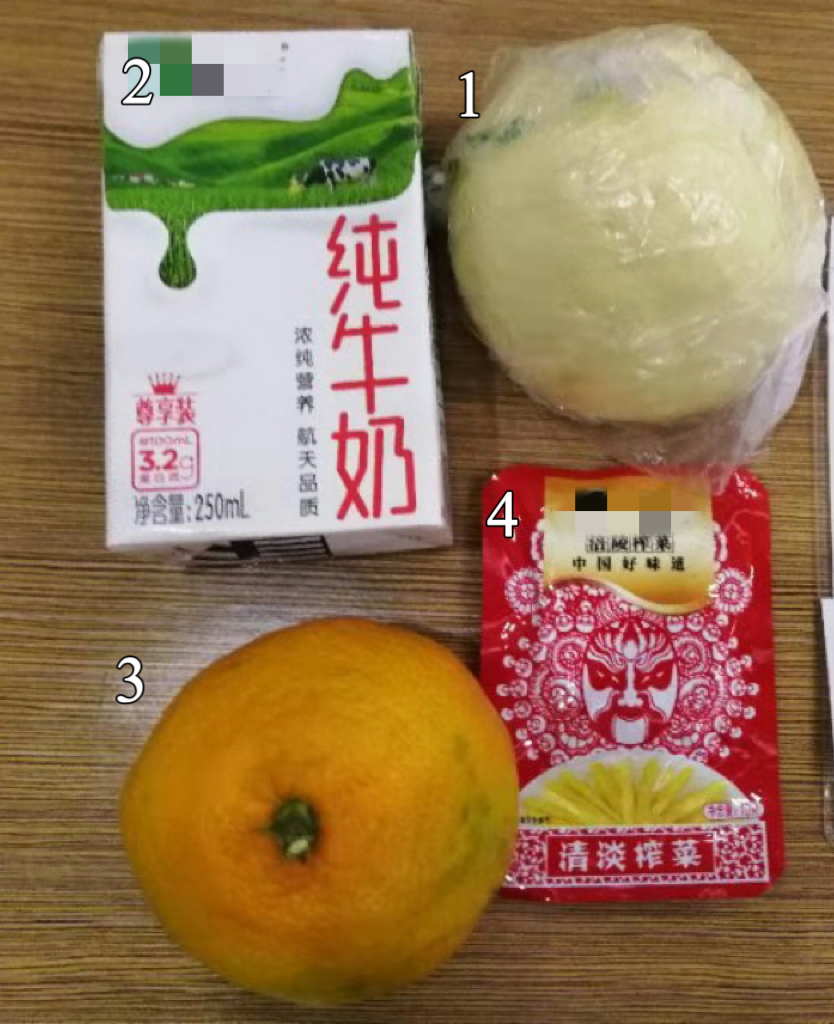Breakfast** | 1 Steamed Corn Bun | Wheat Flour | 60.00 |
|  |  | Yellow Cornmeal | 10.00 |
|  | 2 Milk | Full-fat Milk | 250.00 |
|  | 3 Mandarin Orange | Mandarin Orange | 114.00 |
|  | 4 Pickled Vegetables | Mustard Tuber | 15.00 |
| **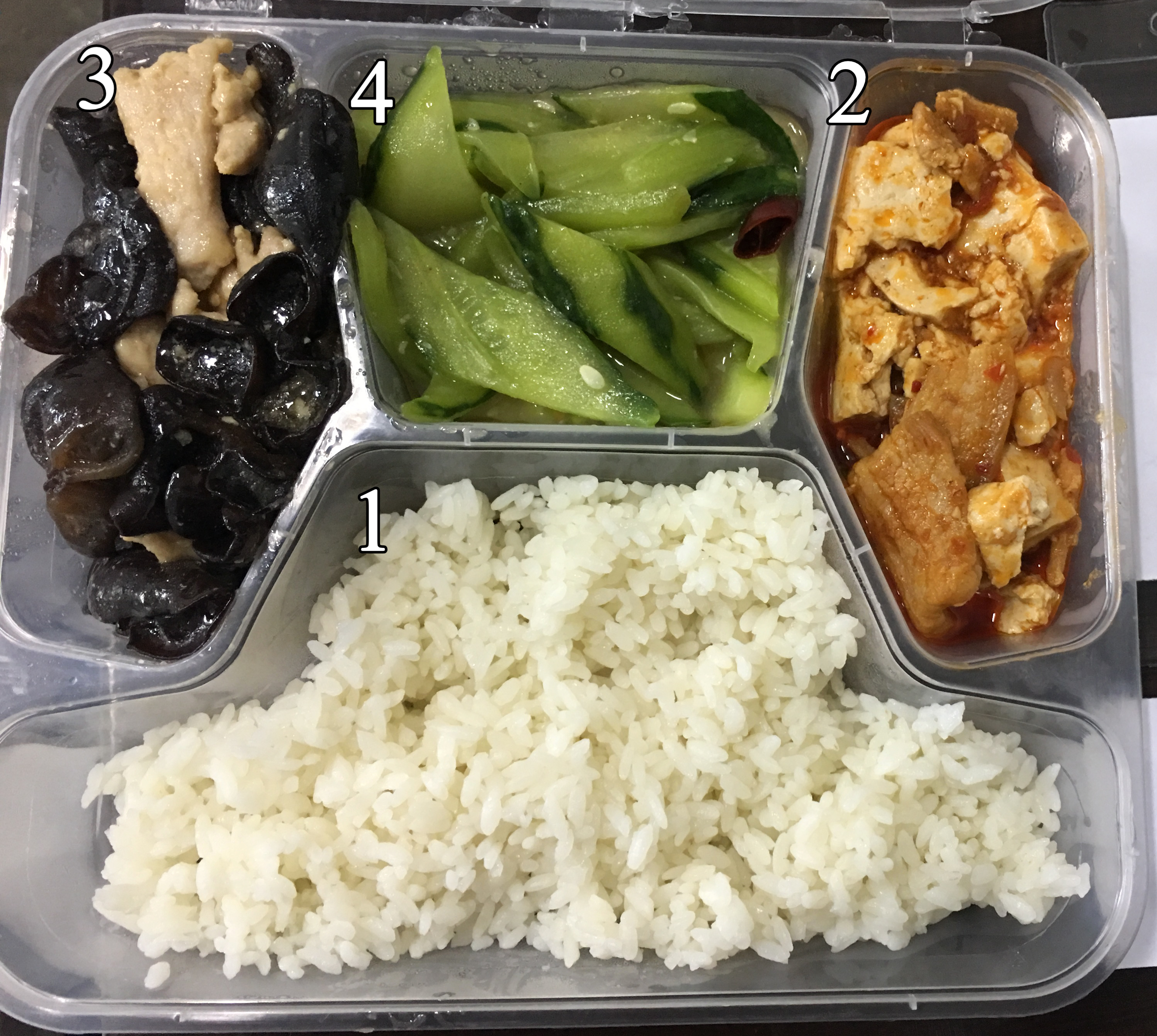Lunch** | 1 Rice | Rice | 90.00 |
|  | 2 Homestyle Tofu | Pork Belly | 34.00 |
|  |  | Tofu | 85.00 |
|  |  | Bean Paste in Chili Oil | 6.00 |
|  |  | Soybean Oil | 4.00 |
|  |  | Corn Starch | 1.53 |
|  |  | Rock Salt | 1.00 |
|  |  | Chicken Powder Seasoning | 0.60 |
|  |  | Soy Sauce | 2.00 |
|  | 3 Stir Fried Pork Slices | Pork Tenderloin | 40.00 |
|  |  | Fungus (dry) | 6.00 |
|  |  | Rock Salt | 1.50 |
|  |  | Soy Sauce | 3.00 |
|  |  | Chicken Powder Seasoning | 0.60 |
|  |  | Pickled Red Pepper | 5.00 |
|  |  | Corn Starch | 2.00 |
|  |  | Soybean Oil | 12.45 |
|  | 4 Stir-fry Cucumber | Cucumber | 138.00 |
|  |  | Rock Salt | 1.40 |
|  |  | Corn Starch | 0.54 |
|  |  | Chicken Powder Seasoning | 0.60 |
|  |  | Lard | 5.00 |
| **Dinner**  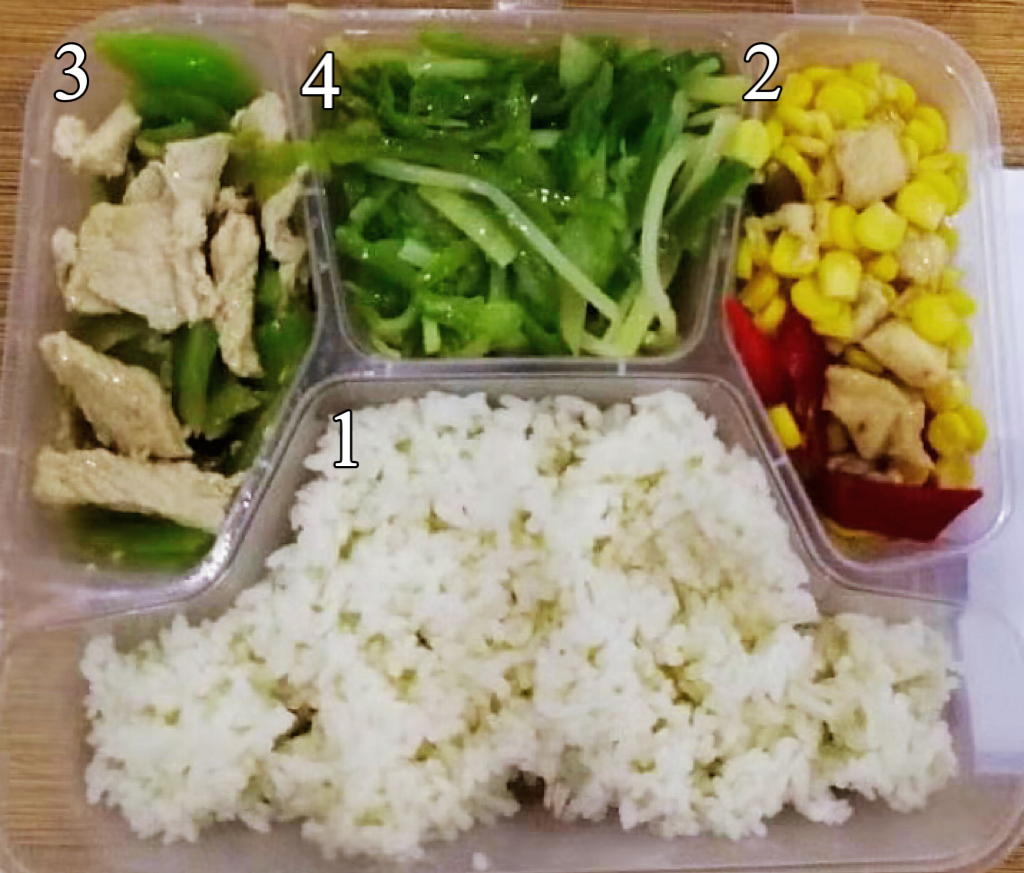 | 1 Rice | Rice | 90.00 |
|  | 2 Diced Chicken with Corn | Drumstick | 41.00 |
|  |  | Corn (fresh) | 69.00 |
|  |  | Rock Salt | 1.30 |
|  |  | Chicken Powder Seasoning | 0.60 |
|  |  | Soybean Oil | 5.00 |
|  |  | Pickled Red Pepper | 5.00 |
|  |  | Corn Starch | 1.40 |
|  | 3 Sautéed Pork Slices with Asparagus Lettuce | Pork Tenderloin | 40.35 |
|  |  | Asparagus Lettuce | 74.00 |
|  |  | Rock Salt | 0.80 |
|  |  | Chicken Powder Seasoning | 0.60 |
|  |  | Soy Sauce | 2.00 |
|  |  | Corn Starch | 2.00 |
|  |  | Soybean Oil | 6.00 |
|  | 4 Potatoes Slivers with Green Chilies | Sweet Pepper | 66.00 |
|  |  | Potato | 47.50 |
|  |  | Corn Starch | 0.55 |
|  |  | Soybean Oil | 6.00 |
|  |  | Rock Salt | 1.30 |
|  |  | Chicken Powder Seasoning | 0.60 |

**Table S2** A quantitative sample menu of Control-SC group in 4-week intervention period (2150 kcal/d)

| Meals | Dishes | Ingredients | Weights (g) |
| --- | --- | --- | --- |
| **Breakfast**  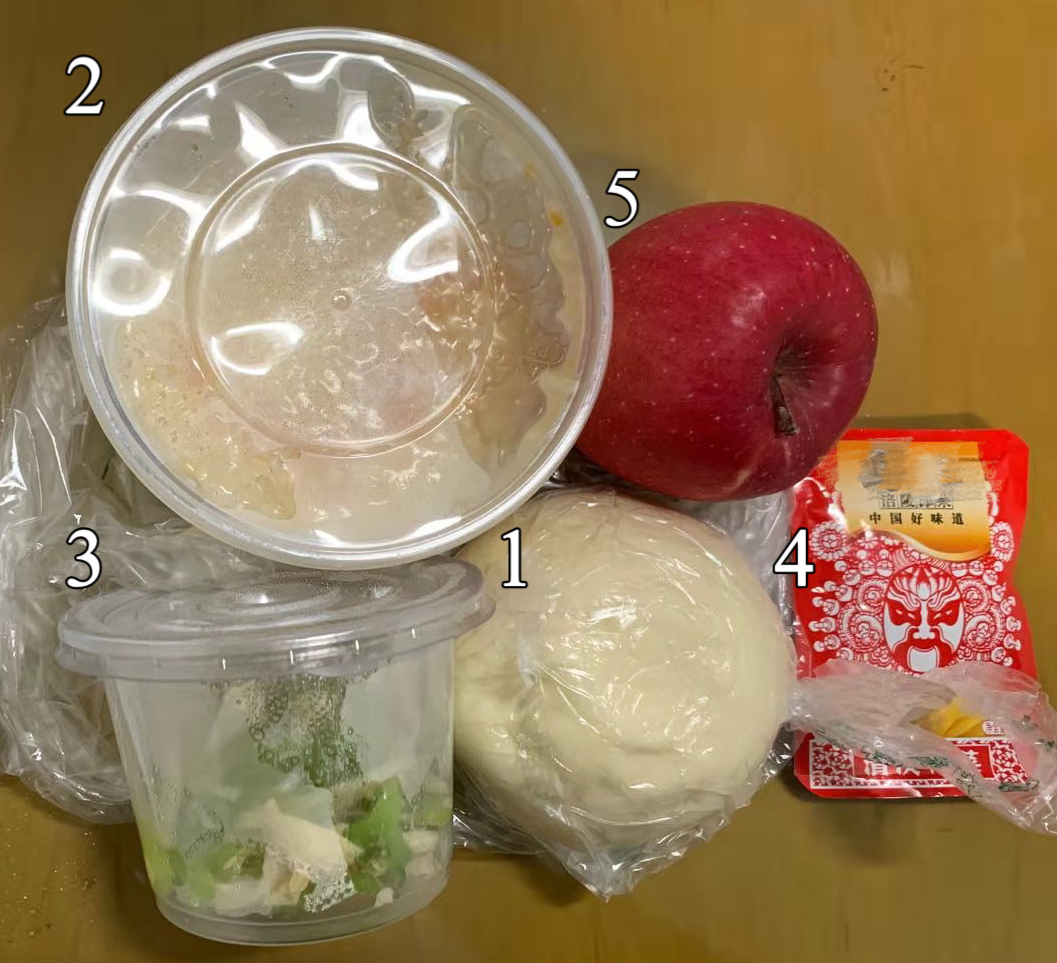 | 1 Steamed Bun | Wheat Flour | 50.00 |
|  | 2 Pumpkin Congee with Millet | Millet | 20.00 |
|  |  | Pumpkin | 33.89 |
|  | 3 Abalone Mushrooms with Asparagus Lettuce and Tofu Skin | Abalone Mushrooms | 30.56 |
|  |  | Asparagus Lettuce | 31.67 |
|  |  | Tofu Skin | 5.00 |
|  |  | Soybean Oil | 5.00 |
|  |  | Chicken Powder Seasoning | 0.60 |
|  |  | Corn Starch | 0.40 |
|  |  | Rock Salt | 0.70 |
|  | 4 Pickled Vegetables | Mustard Tuber | 15.00 |
|  | 5 Apple | Apple | 140.00 |
| **Lunch**  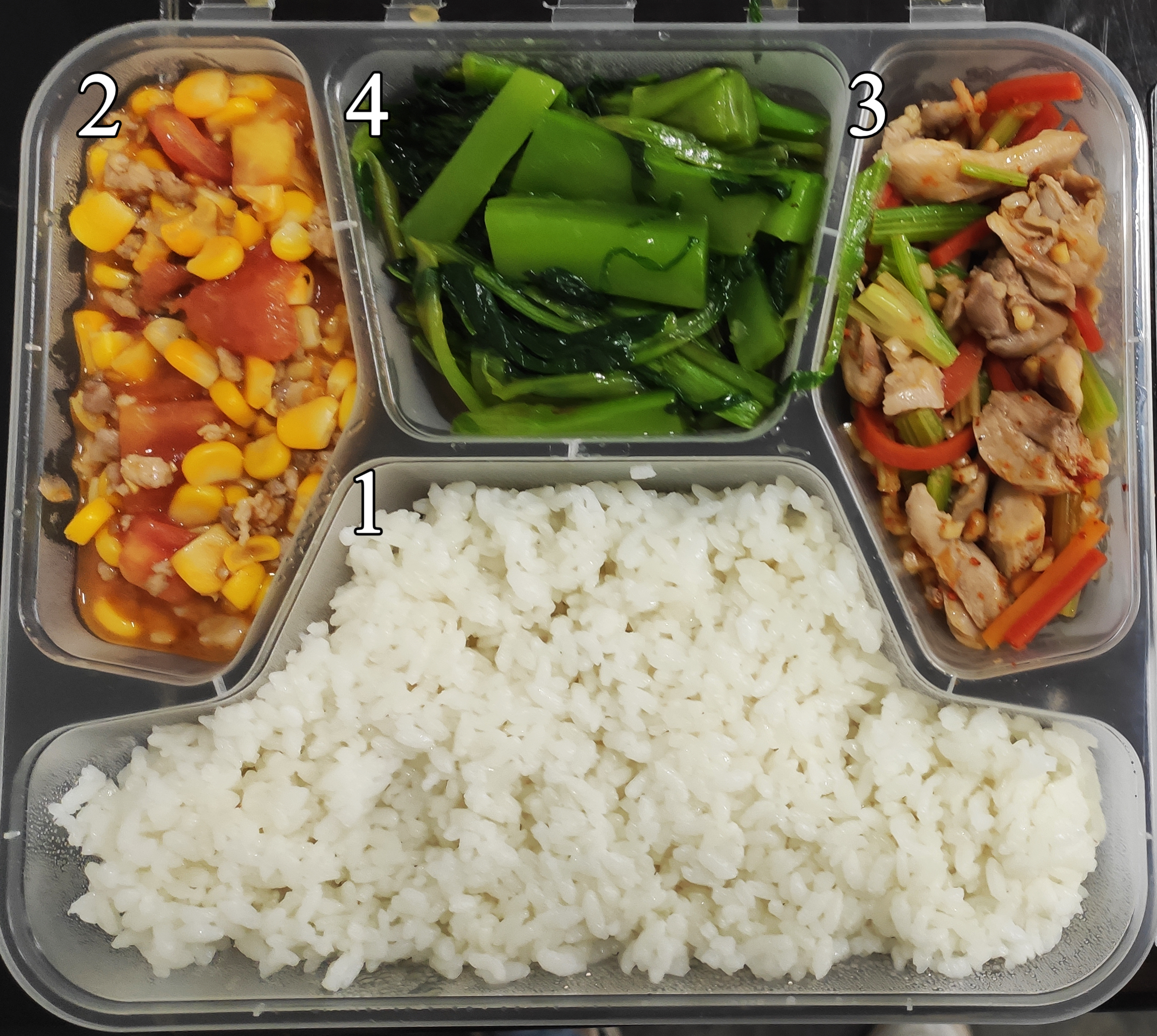 | 1 Rice | Rice | 100.00 |
|  | 2 Multi-ingredient Braising Pork Filling and Corns with Tomato Sauce Flavor | Corn (fresh) | 40.00 |
|  |  | Tomato | 50.67 |
|  |  | Pork Belly | 40.28 |
|  |  | Soybean Oil | 6.00 |
|  |  | Corn Starch | 1.28 |
|  |  | Chicken Powder Seasoning | 0.60 |
|  |  | Soy Sauce | 3.00 |
|  |  | Rock Salt | 0.69 |
|  | 3 Hand-Shredded Chicken | Drumstick | 55.00 |
|  |  | Peanut Kernel (fried) | 8.00 |
|  |  | Sesame Oil | 6.00 |
|  |  | Carrot | 20.00 |
|  |  | Celery Stem | 17.22 |
|  |  | Rock Salt | 0.78 |
|  |  | Chicken Powder Seasoning | 0.60 |
|  |  | Sugar | 1.00 |
|  |  | Soy Sauce | 3.00 |
|  | 4 Scorching Stir Frying Green Vegetables | Lettuce Leaves | 156.11 |
|  |  | Lard | 5.00 |
|  |  | Soy Sauce | 3.00 |
|  |  | Corn Starch | 2.00 |
|  |  | Chicken Powder Seasoning | 0.60 |
|  |  | Rock Salt | 1.17 |
| **Dinner**  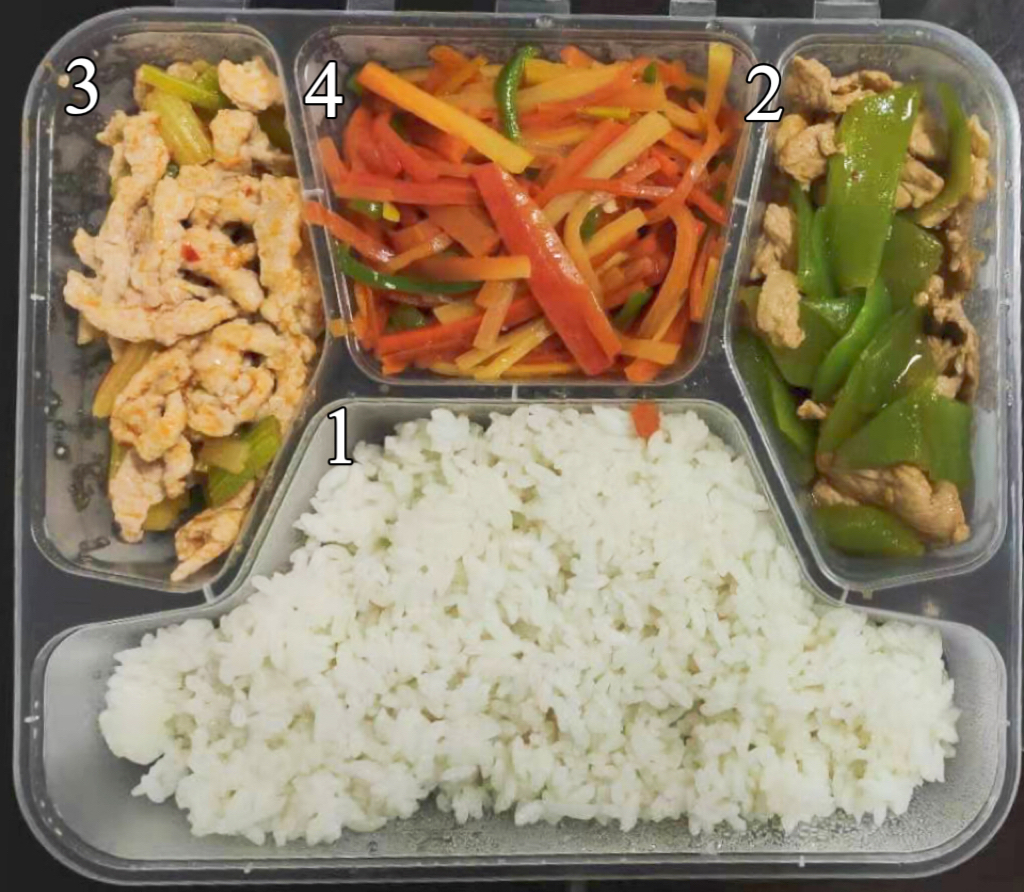 | 1 Rice | Rice | 100.00 |
|  | 2 Frying Pork Slices with Lettuce | Asparagus Lettuce | 55.00 |
|  |  | Pork Trotter | 50.00 |
|  |  | Soybean Oil | 5.00 |
|  |  | Rock Salt | 0.86 |
|  |  | Pickled Red Pepper | 2.97 |
|  |  | Chicken Powder Seasoning | 0.60 |
|  |  | Dark Soysauce | 0.50 |
|  |  | Corn Starch | 0.50 |
|  | 3 Frying Pork Slivers with Celery | Celery Stem | 35.28 |
|  |  | Pork Tenderloin | 50.00 |
|  |  | Soybean Oil | 6.00 |
|  |  | Chicken Powder Seasoning | 0.60 |
|  |  | Rock Salt | 0.80 |
|  |  | Bean Paste in in Chili Oil | 2.00 |
|  |  | Corn Starch | 1.83 |
|  | 4 Carrot with Sweet Pepper | Carrot | 97.22 |
|  |  | Lard | 7.00 |
|  |  | Chicken Powder Seasoning | 0.60 |
|  |  | Soy Sauce | 3.00 |
|  |  | Sweet Pepper | 18.33 |
|  |  | Rock Salt | 1.50 |
|  |  | Corn Starch | 0.67 |

**Table S3** A quantitative sample menu of CHH-SC group in 4-week intervention period (2050 kcal/d)

| Meals | Dishes | Ingredients | Weights (g) |
| --- | --- | --- | --- |
| **Breakfast**  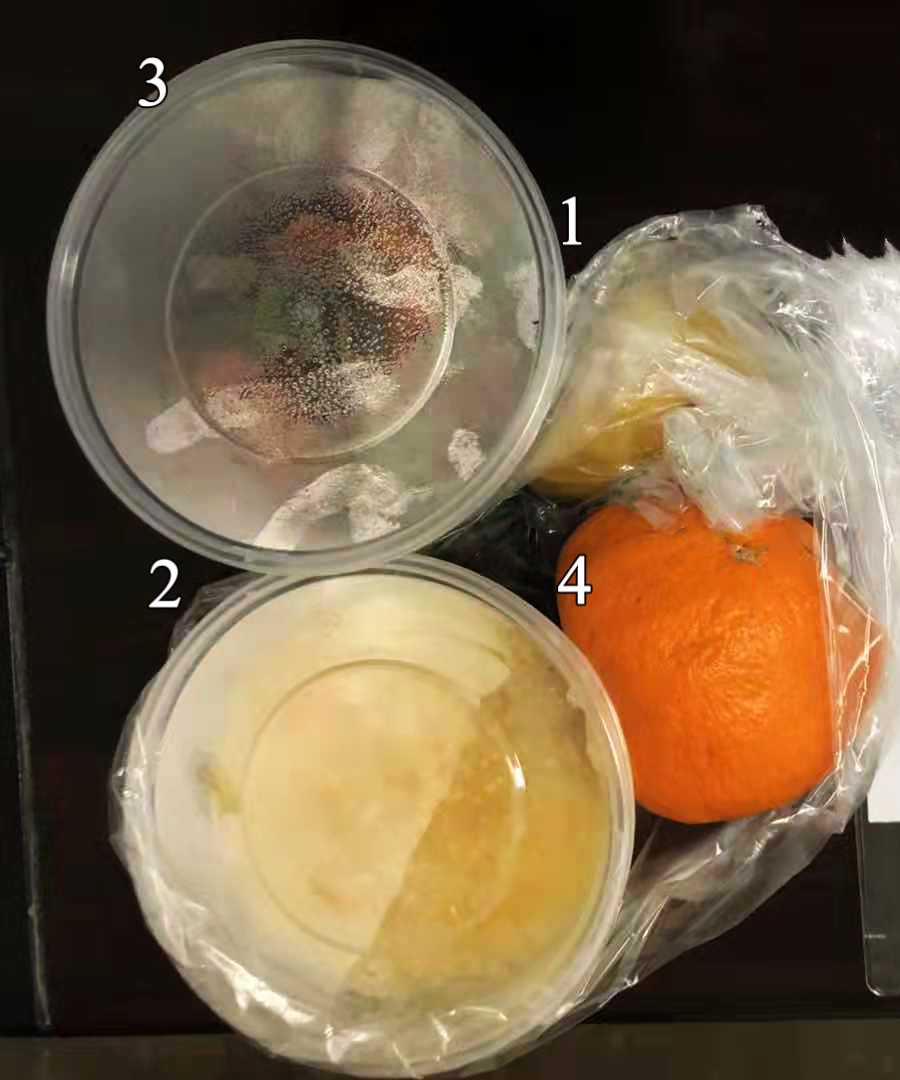 | 1 Creamy Wowotou | Yellow Cornmeal | 40.00 |
|  |  | Wheat Flour | 20.00 |
|  |  | Jujube (dry) | 6.00 |
|  |  | Full-fat Milk | 25.00 |
|  | 2 Pumpkin Congee with Millet | Millet | 20.00 |
|  |  | Pumpkin | 34.06 |
|  | 3 Mushrooms with Asparagus Lettuce and Carrot | Mushroom (dried) | 6.11 |
|  |  | Carrot | 50.00 |
|  |  | Asparagus Lettuce | 30.00 |
|  |  | Sesame Oil | 5.00 |
|  |  | Low-sodium Salt | 1.00 |
|  | 4 Mandarin Orange | Mandarin Orange | 158.00 |
| **Lunch**  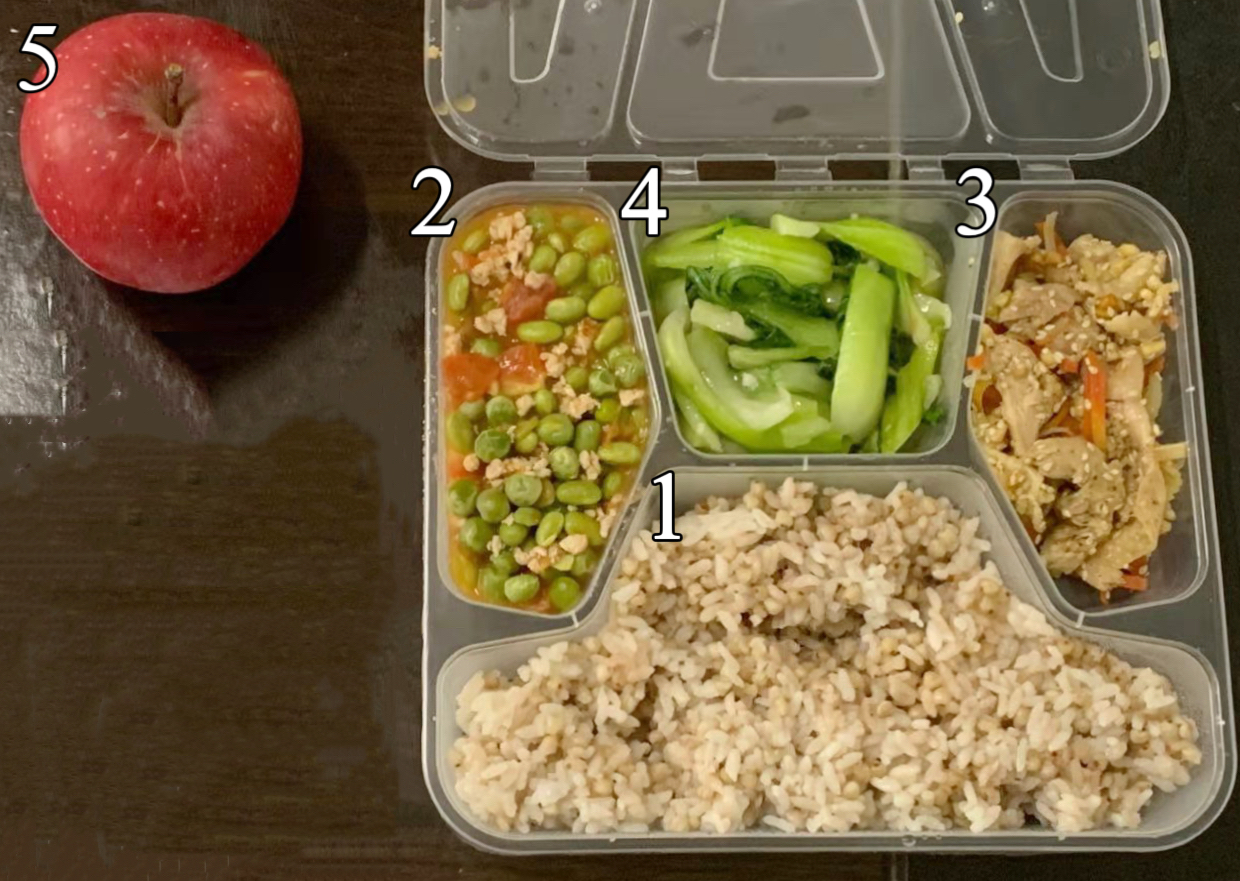 | 1 Grain Rice | Rice | 60.00 |
|  |  | Buckwheat | 30.00 |
|  | 2 Multi-ingredient Braising Pork Filling and Green Beans with Tomato Sauce Flavor | Green Beans | 52.78 |
|  |  | Tomato | 47.44 |
|  |  | Pork Tenderloin | 30.00 |
|  |  | Soybean Oil | 4.00 |
|  |  | Corn Starch | 1.00 |
|  |  | Low-sodium Salt | 1.20 |
|  | 3 Mild Spicy Chicken | Drumstick | 50.28 |
|  |  | Peanut Kernel (fried) | 8.00 |
|  |  | Celery Stem | 10.00 |
|  |  | Carrot | 10.00 |
|  |  | Sesame (white) | 5.00 |
|  |  | Sesame Oil | 2.00 |
|  |  | Sugar | 2.00 |
|  |  | Soy Sauce | 1.00 |
|  |  | Low-sodium Salt | 0.40 |
|  | 4 Scorching Stir Frying Green Vegetables | Brassica Plant | 143.89 |
|  |  | Soybean Oil | 4.00 |
|  |  | Low-sodium Salt | 0.60 |
|  |  | Corn Starch | 0.60 |
|  | 5 Apple | Apple | 147.00 |
| **Dinner**  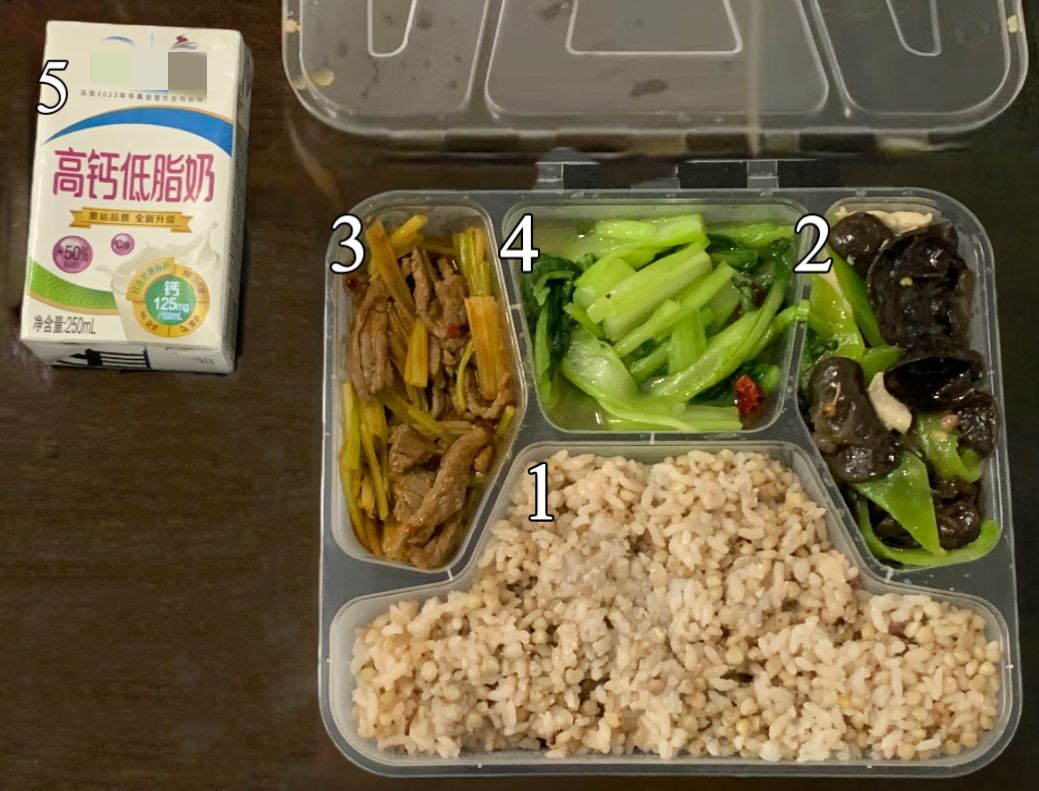 | 1 Grain Rice | Buckwheat | 30.00 |
|  |  | Rice | 60.00 |
|  | 2 Stir Fried Pork Slices | Asparagus Lettuce | 43.06 |
|  |  | Fungus (dry) | 5.00 |
|  |  | Pork Tenderloin | 40.00 |
|  |  | Soybean Oil | 6.00 |
|  |  | Low-sodium Salt | 1.20 |
|  |  | Pickled Red Pepper | 5.00 |
|  |  | Corn Starch | 2.50 |
|  | 3 Frying Beef Slices with Celery | Celery Stem | 34.17 |
|  |  | Beef Tenderloin | 40.00 |
|  |  | Soybean Oil | 5.00 |
|  |  | Bean Paste in in Chili Oil | 2.00 |
|  |  | Soy Sauce | 2.00 |
|  |  | Low-sodium Salt | 0.30 |
|  |  | Corn Starch | 1.81 |
|  | 4 Chinese Cabbage with garlic flavor | Chinese Cabbage | 131.11 |
|  |  | Soybean Oil | 3.00 |
|  |  | Corn Starch | 1.00 |
|  |  | Low-sodium Salt | 1.00 |
|  | 5 Milk | High-calcium and Low-fat Milk | 250.00 |
